# Research Progress on the Immune Function of Liver Sinusoidal Endothelial Cells in Sepsis

**DOI:** 10.3390/cells14050373

**Published:** 2025-03-04

**Authors:** Xinrui Wang, Zhe Guo, Yuxiang Xia, Xuesong Wang, Zhong Wang

**Affiliations:** 1 School of Clinical Medicine, Tsinghua University, Beijing 100190, China; wangxinrui1405@163.com (X.W.); chenjiu0525@163.com (Y.X.); 2 Beijing Tsinghua Changgung Hospital, Tsinghua University, Beijing 100084, China; gza01482@btch.edu.cn

**Keywords:** sepsis, liver sinusoidal endothelial cell, organ dysfunction, clearance, immune function

## Abstract

Sepsis is a complex clinical syndrome closely associated with the occurrence of acute organ dysfunction and is often characterized by high mortality. Due to the rapid progression of sepsis, early diagnosis and intervention are crucial. Recent research has focused on exploring the pathological response involved in the process of sepsis. Liver sinusoidal endothelial cells (LSECs) are a special type of endothelial cell and an important component of liver non-parenchymal cells. Unlike general endothelial cells, which mainly provide a barrier function within the body, LSECs also have important functions in the clearance and regulation of the immune response. LSECs are not only vital antigen-presenting cells (APCs) in the immune system but also play a significant role in the development of infectious diseases and tumors through their specific immune regulatory pathways. However, in certain disease states, the functions of LSECs may be impaired, leading to immune imbalance and the development of organ failure. Investigating the immune pathways of LSECs in sepsis may provide new solutions for the prevention and treatment of sepsis and is crucial for maintaining microcirculation and improving patient outcomes.

## 1. Background

Sepsis is characterized as a life-threatening condition due to organ dysfunction caused by an abnormal host response to infection. This condition represents a complex clinical syndrome response to infection [1]. Sepsis impacts around 50 million individuals globally, leading to more than 11 million fatalities, which constitute approximately 20% of all deaths worldwide. Even survivors of the disease often suffer from long-term complications, significantly reducing their quality of life and posing a substantial threat to human health [2]. Due to the rapid progression of sepsis, it becomes extremely challenging to repair damaged organ functions once they occur; thus, early detection and intervention are essential [3]. Recent research has focused on exploring the pathological processes of sepsis, with the hope of intervening in the early stages of the disease to improve treatment outcomes and patient prognosis. The immune system plays a critical role throughout sepsis development. During the initial stage of pathogen invasion, the immune system is activated, leading to the release of numerous cytokines. Multiple immune mechanisms work synergistically to combat the pathogens. However, in the terminal stage of the disease, dysfunction and deregulation of humoral and cellular immune responses may be the primary reasons for the progression of infection and the occurrence of various comorbid infections [4,5]. When the immune barrier is impaired, invading pathogens and opportunistic pathogens in the body can become pathogenic factors, causing damage. Endotoxins and various cytokines can affect capillary endothelial cells, leading to their loss of function and ultimately resulting in multiple organ failure [6,7]. The liver is a vital organ in the human body, receiving 25% of the blood supply from the heart. Due to its abundant blood supply, the liver plays an essential and unique role in both the physiological and pathological processes of the body, making it a vital organ that impacts the survival rates of sepsis patients [8,9]. In recent years, there has been significant progress in understanding the liver’s immune regulation and endocrine functions.

In clinical studies related to sepsis and septic shock, attention is often paid to vital organs such as the heart, kidney, and brain, particularly the kidney’s filtration and clearance functions, with special emphasis on maintaining renal perfusion pressure during early hemodynamic disturbances. However, the role of the liver is frequently overlooked. As a crucial immunomodulatory organ, various cells in the liver are involved in the body’s response processes, maintaining microvascular perfusion levels and preventing ischemia–reperfusion injury [10,11]. Enhancing liver blood supply and preserving its basic physiological functions can significantly improve the prognosis of sepsis patients. As our understanding of sepsis deepens, research in these areas is also progressing. Many aspects of the scavenger receptor mechanism in LSECs still require further investigation and validation.

LSECs are non-parenchymal cells, making up approximately 3.3% of the total cell population in the liver. As a specialized type of endothelial cell found in the liver, LSECs are primarily located in the walls of liver sinusoids. Despite accounting for a small proportion in the liver, LSECs and Kupffer cells (KCs) are crucial contributors in various liver reactions and regulatory functions, exerting specific roles in immune processes [12,13]. LSECs play a vital role in the body’s infection response due to their unique structural characteristics, such as large intercellular gaps and the absence of a basement membrane [14,15]. During infection, pathogens release endotoxins into the bloodstream. When this endotoxin-containing blood flows through the hepatic sinusoids along with inflammatory factors released by other tissue cells, it passes through the gaps between LSECs, facilitating broad contact and interaction with parenchymal and non-parenchymal liver cells. Studies on LSEC function have demonstrated their high endocytic receptor activity, which is crucial for their clearance capacity [16]. LSECs possess a potent ability to clear physicochemical components from the bloodstream. Recent studies have indicated that LSECs also play a crucial role in eliminating immune complexes and viruses generated during the human immune response [17]. As a vital non-parenchymal cell type in the liver, the functions of LSECs extend beyond the filtration and clearance of blood. These cells, endowed with immune capabilities, serve a pivotal role in immune responses. During immune reactions, LSECs can influence various stages of the immune process through regulatory pathways on their cell membranes [18,19]. In sepsis, endotoxins released into the bloodstream can cause damage and necrosis to LSECs, leading to increased oxidative stress and the emergence of subsequent related reactions. As important participants in immunity, LSECs engage in both humoral and cellular immune responses during infection through multiple direct and indirect pathways, thereby participating in the immune response. By expressing various adhesion molecules, LSECs mobilize immune cells to the liver, indirectly influencing cellular immunity. Furthermore, with advancing research, the mechanisms underlying the interactions between LSECs and various immune cells are gradually being elucidated.

## 2. The Clearance Function of LSECs

### 2.1. Direct Clearance Function

**Figure 1:**
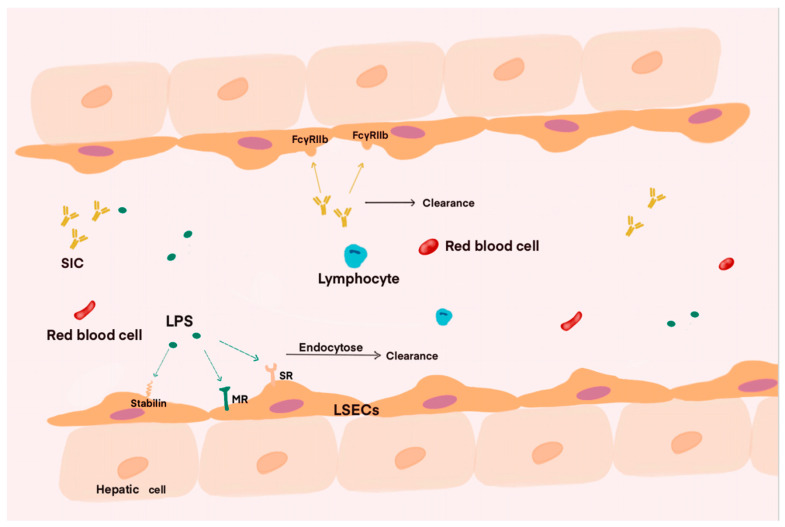
The scavenging function of liver sinusoidal endothelial cells plays a crucial role during infection. As blood flows through the liver sinusoids, substances such as antigen–antibody complexes and LPS can be cleared by the action of these cells. Specifically, the clearance of LPS relies on endocytosis receptors, such as stabilin, SR, and MR, while antigen–antibody complexes and viruses are removed via corresponding receptors during their passage through the liver sinusoids. Consequently, the content of LPS, SIC, and other related substances in the blood decreases significantly after flowing through the liver sinusoids.

Unlike other endothelial cells, the unique structure of LSECs enable them to facilitate the free passage of macromolecules, including colloidal components. Studies have indicated that LSECs possess highly active receptors. These characteristics make them one of the most effective scavenger cells in the body [20] (Figure 1). During the course of sepsis, various physicochemical factors released by pathogens affect the human body. A clinically relevant study based on endotoxin in septic patients showed that many patients exhibited higher endotoxin activity in their plasma upon admission to the ICU. The presence of endotoxemia also indicates a poor prognosis for the patient [21,22]. Therefore, clearing endotoxins, or lipopolysaccharide (LPS), that enter the circulation may be one of the effective measures to improve endotoxemia. Both LSECs and KCs in the liver are capable of exerting scavenging functions through their own mechanisms when LPS are released into the bloodstream. Conventionally, it is believed that KCs, as the resident macrophages in the liver, may play a primary role in clearing LPS from the blood. Previous research by Shao has demonstrated that KCs uptake LPS in the hepatic blood through phagocytosis [23]. However, as research progresses on the clearance of extracellular matrix components and macromolecules such as LPS, it has been discovered that the elimination of soluble macromolecules and nanoparticles is primarily mediated by LSECs via endocytosis [24]. The endocytic receptors of LSECs mainly include scavenger receptor (SR) [25], stabilin-1, stabilin-2 [26], and mannose receptor (MR) [27,28]. The scavenger receptors mentioned above can bind to LPS and transport it to lysosomes for clearance through endocytosis. Among various endocytic receptors, the stabilin receptor plays a major role in influencing LPS clearance and the endocytic function of LSECs. Furthermore, among the two receptor forms, stabilin-1 is a crucial factor affecting LPS clearance and performs a vital regulatory function. This viewpoint has been confirmed by the previous research [29,30].

The scavenging function of LSECs is not limited to clearing physicochemical components from the blood. Studies have shown that LSECs also play a role in eliminating viruses and immune complexes generated during immune responses [31,32,33]. Ganesan and colleagues discovered that LSECs express FcγRIIb as their only Fcγ receptor, through which LSECs could eliminate blood-derived small immune complexes (SICs), thereby controlling autoimmune diseases [34]. The discovery that LSECs can clear blood-derived phages through endocytosis provides a solution for subsequent research on related diseases [35,36]. Apart from expressing various endocytic receptors and playing a role in clearance, LSECs also participate in the body’s immune response through their unique mechanisms. By exploring and intervening in the immune response mediated by LSECs, new directions may be provided for the treatment of patients with sepsis. As our understanding of sepsis continues to deepen, research in these areas is also advancing, and there are still many aspects of the clearance mechanism of liver sinusoidal endothelial cells that require further investigation and validation.

### 2.2. Lipoprotein-Related Clearance

The clearance of 75% of the LPS in the body is associated with LSECs. The elimination of LPS is a complex process. Besides the phagocytosis of KCs and the endocytosis receptor-mediated clearance of LSECs, LSECs can also promote the efficient removal of LPS from plasma through high-density lipoprotein (HDL) [37]. The findings of the study demonstrate that the concentration of HDL in blood plasma is associated with the rate of LPS elimination. Acting as a carrier for LPS, HDL forms an LPS–HDL complex, which has the ability to bind to stabilizing proteins 1 and 2. This binding allows for intracellular transportation to lysosomes, ultimately aiding in the elimination of LPS from the body [38]. Apart from this, Ganesan et al. have confirmed that the scavenger receptor B1, highly expressed in mouse LSECs, may be involved in HDL-mediated clearance by LSECs [39].

In addition to directly clearing substances, LSECs also indirectly exert their scavenging function through relevant carriers during the immune and clearance processes. In a review article by Liu et al., the researchers summarized the common pathogenic bacteria causing bloodstream infections in liver transplant patients from previous studies, among which Pseudomonas aeruginosa accounted for a significant proportion. The reason may be attributed to the drug resistance of Pseudomonas aeruginosa to common antibiotics. Furthermore, retrospective analysis results from Kim et al. indicated that the primary source of Pseudomonas aeruginosa in patients with bacteremia after liver transplantation was the biliary tract [40,41]. Studies have shown that LSECs infected with Pseudomonas aeruginosa exhibit significant ultrastructural changes, such as thinning of the endothelium and reduced porosity. This may lead to the loss of pore structure in LSECs, thereby affecting their clearance- and immune-related functions [42]. In humans, similar structural changes may occur in LSECs during severe infections. The loss of fenestrations in LSECs impairs the liver’s ability to take up lipoproteins and chylomicrons, leading to subsequent hyperlipidemia [43] and insulin resistance [44]. This underscores the critical role of LSECs in sepsis-related lipoprotein lipase inhibition and increased hepatic triglyceride transport.

## 3. Enhanced Platelet Adhesion

Multiple factors can lead to the impairment of organ barrier function, triggering a series of inflammatory responses. When the liver is damaged, LSECs may detach from the hepatic sinusoidal wall due to ischemia or other factors and adhere to platelets in the blood, resulting in the interaction between LSECs and platelets [45,46]. Meyer’s research findings indicate that contact between LSECs and platelets induces the secretion of hepatocyte growth factor by hepatic stellate cells, thereby enhancing the proliferation of hepatocytes [46]. Furthermore, cellular experiment results related to LSECs also confirm that the interaction between LSECs and platelets can promote the production of IL-6 and vascular endothelial growth factor, which similarly induce the proliferation of hepatocytes [45]. During organ ischemia–reperfusion, an increase in platelet count contributes to the repair of damaged organs, ensuring the function of vital organs. Maintaining the function of LSECs is crucial for sustaining immune responses and repairing subsequent organ damage, as they are key drivers for enhancing platelet activation and adhesion.

## 4. The Role of LSECs in Immune Responses

LSECs play a critical role in the hepatic immune response by participating in immune regulation and protecting liver tissue from damage. By influencing the filtration and modulation at the blood–liver interface, LSECs help maintain immune homeostasis through regulating sinusoidal blood flow, promoting the interaction between immune cells and blood components. They also exert anti-inflammatory and antioxidant effects by expressing various anti-inflammatory factors and modulating the expression of cell adhesion molecules, thereby mitigating inflammatory responses.

LSECs produce immune-suppressive molecules, such as programmed death ligand-1(PD-L1), to inhibit T cell activity and prevent excessive immune responses [47]. Additionally, LSECs can capture and present antigens, activating immune cells and mediating immune responses [48]. Through interactions with immune cells, such as KCs and T cells, LSECs regulate immune cell activity and maintain immune balance [49,50]. By promoting the production of anti-inflammatory cytokines by immune cells, LSECs contribute to inducing immune tolerance, modulating excessive immune responses, and reducing immune-mediated damage [51]. In summary, LSECs play a crucial role in maintaining liver immune balance, defending against inflammation, and protecting liver tissue from damage. These mechanisms help prevent the progression of chronic inflammation, fibrosis, and liver disease. However, in certain pathological conditions, the functions of LSECs may be impaired, leading to immune imbalance and the development of liver diseases.

### 4.1. The Role of LSECs in Acute Inflammation

LSECs maintain close contact with the blood flowing through the liver, rapidly eliminating pathogenic substances such as LPS, performing scavenging functions, and exerting immune functions. As specialized immune cells, LSECs play a crucial role in acute inflammation through multiple mechanisms. These pathways are interconnected and interact with each other, constituting the primary immune barrier of LSECs.

#### 4.1.1. Antigen Presentation

Under normal conditions, although LSECs play a role in regulating immune function, their expression level of major histocompatibility complex class II (MHC II) is relatively low, and they lack the ability to activate T cells. Therefore, in typical immune regulation processes, their role as immune cells is not prominent [52].

Conventionally, antigen-presenting cells such as dendritic cells and B cells can ingest and process antigens, presenting them to T cells and activating the immune process. However, there are differences between the antigen-presenting function of LSECs and traditional antigen-presenting cells. As specialized endothelial cells, LSECs can capture antigens in the blood through specific receptors on their cell surface while being exposed to blood. The primary receptors involved include pattern recognition receptors (PRRs), SR, and mannose receptors [53,54]. In the classical immune process, MHC-I molecules primarily present endogenous antigens. In the liver, LSECs, through cross-presentation, load captured exogenous antigens (such as viral antigens) onto MHC-I molecules, further activating CD8+ T lymphocytes [55]. This process contributes to the clearance of pathogens and other substances in the blood, playing a role in immunity. Additionally, CD1d molecules are also expressed in LSECs. CD1d is the main recognition molecule for natural killer T cells, presenting lipid and glycolipid antigens [56,57]. When stimulated by pathogens such as LPS or viruses, LSECs capture glycolipid antigens from the blood. The expression of CD1d and antigen presentation are enhanced through cellular pathways [58], forming CD1d–antigen complexes in the body. LSECs transport these complexes to the cell membrane surface, activating NKT cell priming.

#### 4.1.2. Spatial Polarization

In the early stages of the inflammatory response, LSECs closely interact with pathogens and their metabolites within the liver sinusoids. The study by Gola and colleagues confirms the role of the myeloid differentiation primary response 88 (MYD88) pathway in the immune function of LSECs. Under physiological conditions, pathogens induce persistent MYD88-dependent signaling, enabling LSECs to regulate the formation of chemokines and the surrounding extracellular matrix. This regulation affects the spatial distribution of immune cells, leading to immune spatial polarization. This creates a refined area around the portal vein, restricting bacterial activity and preventing further spread of pathogens [59]. From this perspective, LSECs not only serve as antigen-sensing cells but also detect microorganisms upon antigen exposure. They respond swiftly by coordinating the localization of immune cells, thus establishing spatial polarization. This process enhances the host’s defense mechanisms and reduces the damage caused by infection [60,61,62].

#### 4.1.3. Cell Recruitment

**Figure 2:**
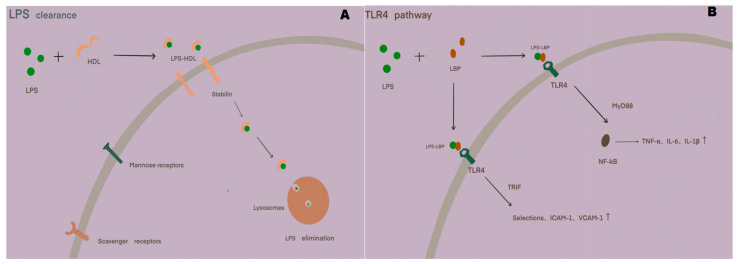
(**A**) Schematic diagram of LPS clearance: LPS entering the systemic circulation binds to HDL in the blood to form an LPS–HDL complex. This complex circulates through the bloodstream and binds to endocytosis receptors on the surface of LSECs, primarily stabilized proteins. It is then transported intracellularly to lysosomes, where the LPS clearance effect is exerted. (**B**) Schematic diagram of the TLR4 signaling pathway: LPS entering the systemic circulation binds to LBP in the blood to form a complex. This complex interacts with TLR4 receptors on the surface of LSECs, activating the signaling pathway. When the MYD88 pathway is activated, it interacts with the downstream NF-KB pathway, promoting the release of TNF-α, IL-6, and IL-1β. When the TRIF pathway is activated, it stimulates the release of proteins such as selectins, ICAM-1, and VCAM-1, influencing the progression of inflammatory responses.

During inflammation, LSECs control the synthesis and expression of various adhesion molecules, promoting the recruitment and accumulation of leukocytes and lymphocytes in the liver [50,63]. When exposed to pathogens and their metabolites, LSECs regulate leukocyte recruitment by differentially expressing adhesion molecules such as intercellular adhesion molecule 1 (ICAM-1) and vascular cell adhesion protein 1 (VCAM-1) [64,65]. Animal studies by Heesch and colleagues on Listeria infection and monocytic proliferation confirmed that LSECs induce the recruitment of CD8+ T lymphocytes in the liver by expressing CXC chemokine ligand 16 (CXCL16), which attracts T cells expressing CXC chemokine receptor 6 (CXCR6) [66]. In research related to viral infections, Bruns and colleagues extracted LSECs from transplanted livers and infected them with cytomegalovirus. The results demonstrated that during this infection, the expression of ICAM-1 and CXC chemokine ligand 10 (CXCL10) increased, leading to the recruitment of CD8+ T lymphocytes to the liver [67]. In addition, a study on LSECs in acute inflammatory liver injury found that by observing the livers of mice injected with LPS, LSECs coordinate the recruitment and activation of nature killer (NK) cells during acute inflammatory liver injury. They also stimulate the secretion of various chemokines and cytokines through NK cells, promoting the recruitment and activation of lymphocytes [68]. LSECs interact with immune cells through various mechanisms, regulating inflammatory responses [69] (Figure 2).

#### 4.1.4. Cytokine Expression

Tumor necrosis factor-α (TNF-α), interleukin (IL)-1β, and IL-6 are cytokines produced by the immune system during the initial stages of infection. These cytokines act on endothelial cells and enter the bloodstream, leading to a series of infection-related symptoms. As mentioned in previous related reviews, the presence of these cytokines, especially IL-6, may provide important clues for the development of sepsis. IL-6 is a multifunctional cytokine, and studies have shown that elevated levels of IL-6 are indicators of sepsis progression and prognosis in patients with severe infections [70,71]. When immune cells and endotoxins interact with LSECs in the hepatic sinusoids, LSECs can sense changes in the levels of endotoxins and other substances in the blood, thereby performing a range of antigen presentation and immune induction functions. When the body is in an inflammatory state, LSECs are stimulated by LPS, the ligand of Toll like receptor 4 (TLR4), leading to increased expression of molecules such as CD54 and CD106 on the cell membrane surface. This stimulation also results in the production of large amounts of IL-6, which participates in the immune response [72]. In patients with liver damage, IL-6 is significantly enriched in LSECs [73]. During liver damage, transforming growth factor-beta 1 (TGF-β1) can also stimulate LSECs to secrete IL-6 [74]. LSECs not only affect IL-6 levels, but kyioannou et al. have demonstrated that LSECs can also regulate IL-12 and IL-18 levels by influencing NK cell levels [63]. NK cells, along with IL-12 and IL-18, play a crucial role in the progression and outcome of sepsis. As key sentinel cells in the immune response, LSECs can sense various pathways and produce different levels of cytokines, thereby influencing the progression of the immune response and the development of related complications.

#### 4.1.5. The PD-L1 Pathway Affects the Progression of the Immune Response in Sepsis

PD-L1, a ligand for the co-inhibitory receptor PD-1, is expressed on B cells, T cells, and myeloid cells. It plays a crucial role in various immune responses [75,76]. The PD-L1 pathway has been associated with the onset and progression of sepsis, leading to sepsis-related immunosuppression. It represents a key target for intervention in septic shock [77]. PD-L1 can inhibit lymphocyte apoptosis and reverse monocyte dysfunction by blocking specific pathways. According to Wang et al., the administration of PD-L1 antibodies to mice with cecal ligation and puncture (CLP) significantly improves their survival rate [78]. LSECs, a unique type of endothelial cell, not only play a role in antigen presentation but also regulate immune responses through PD-L1 expression. During various inflammatory reactions, LSECs express PD-L1, and this expression is significantly upregulated during sepsis [79,80]. The presence of PD-L1 on LSECs may contribute to the pathology of sepsis-associated liver injury, influenced by the interaction between PD-1+ KCs and PD-L1 on LSECs [81]. LSECs can interact with naive CD8+ T cells through the expression of PD-L1, which binds to PD-1 on T cells, thereby promoting immunosuppression. Zhang et al.’s research findings confirm that when septic mice are administered a PD-L1 blocker, their survival rate improves, providing direction for subsequent clinical studies [82]. As an important pathway in sepsis and its complications, PD-L1 deserves further investigation. Exploring the expression of PD-L1 receptors on LSECs remains a highly valuable research area.

#### 4.1.6. Regulatory T Cell Differentiation

LSECs play a dual role in the hepatic microenvironment: filtering blood and regulating immunity. LSECs exert a pivotal function in the modulation of the hepatic immune system through the expression of Glycoprotein A Repetitions Predominant (GARP) [83]. This transmembrane protein is primarily found on regulatory T cells (Tregs) and platelets, but it is also present on LSECs. GARP interacts with and activates the precursor of TGF-β, a multifunctional cytokine that is crucial for immune regulation, cell growth, differentiation, and apoptosis [84]. Within the liver microenvironment, LSECs use GARP to capture circulating TGF-β precursors. GARP then facilitates their conversion into the active form of TGF-β [85]. Activated TGF-β binds to TGF-β receptors on adjacent T cells, triggering various signaling pathways [86,87]. Specifically, the binding of TGF-β to T cell receptors leads to the phosphorylation of Smad proteins, which then migrate to the cell nucleus to regulate gene expression. This pathway is crucial for the differentiation and function of Tregs. Tregs maintain immune homeostasis and self-tolerance by secreting inhibitory cytokines such as IL-10 and TGF-β, and directly suppressing the activation of effector T cells through cell-to-cell contact [88,89].

In the liver, LSECs enhance the generation and function of local Tregs through GARP-mediated TGF-β activation, thereby preventing excessive immune responses [90]. This mechanism is crucial for preventing immune-mediated damage in autoimmune liver diseases and chronic hepatitis. It also provides insights into maintaining immune tolerance after liver transplantation to avoid transplant rejection. By expressing GARP and facilitating the activation of TGF-β, LSECs influence T cell function, especially that of regulatory T cells, creating a complex immune regulatory network within the liver. This mechanism offers new insights into the pathogenesis of liver diseases and presents potential therapeutic targets. For instance, targeting GARP or TGF-β signaling pathways could be highly promising in the treatment of autoimmune liver disorders, chronic hepatitis, or preventing transplant rejection.

LSECs can regulate TGF-β and T cell function through body fluid circulation and affect the whole body. Liu et al. found that the occurrence of pulmonary allergic diseases is related to the interaction between the liver and T cells. In the study, they used nanoparticles to deliver mouse allergen ovalbumin to the liver. The increase of ligands can effectively reduce allergic inflammation in the lungs, increase the production of TGF-β and Tregs, and exert an immunosuppressive effect [91].

#### 4.1.7. Expression of Toll-like Receptor 4 (TLR4) Receptors in LSECs

As TLR4 serve as the signaling receptors for LPS, animal studies have demonstrated that TLR4 receptor downregulation in mice leads to reduced reactivity to LPS, confirming the correlation between TLR4 expression levels and LPS sensitivity [92,93]. Compared to the spleen and lungs, the liver expresses lower levels of TLR4, with the expression primarily localized to LSECs [94]. Various binding proteins in the serum can interact with LPS and TLR4 to exert regulatory effects, among which CD14 and LPS binding protein are key modulators [95]. The formation of a complex between these two binding proteins and LPS, which then binds to TLR4 receptors on the surface of LSECs, activates immune cell sensitivity to LPS and exerts a barrier function [96].

Serving as an important regulatory receptor, TLR4 also plays a specific role in the immune modulation process of LSECs through signaling pathways. The TLR4 signaling pathways primarily include the MYD88-dependent pathway and the TRIF pathway. When the TLR4 pathway on the surface of LSECs is activated by endotoxin-related factors, it binds to MYD88, further activating the NF-kB and MAPK signaling pathways. This, in turn, stimulates the upregulation of the expression levels of corresponding proinflammatory factors such as TNF-α, IL-6, and IL-1β, recruiting immune cells and forming local immunity [97,98,99]. TLR4 can also promote the expression of molecules such as selectins, ICAM-1, and VCAM-1 by binding to TRIF and related proteins, facilitating immune cell adhesion and intensifying inflammatory responses [100,101,102] (Figure 2). In addition, the TLR4 pathway can regulate the permeability of LSECs through related signaling pathways, affecting their structural function and producing corresponding pathological effects [103].

#### 4.1.8. Influence of FABP4 on Metabolism and Inflammatory Responses

**Figure 3:**
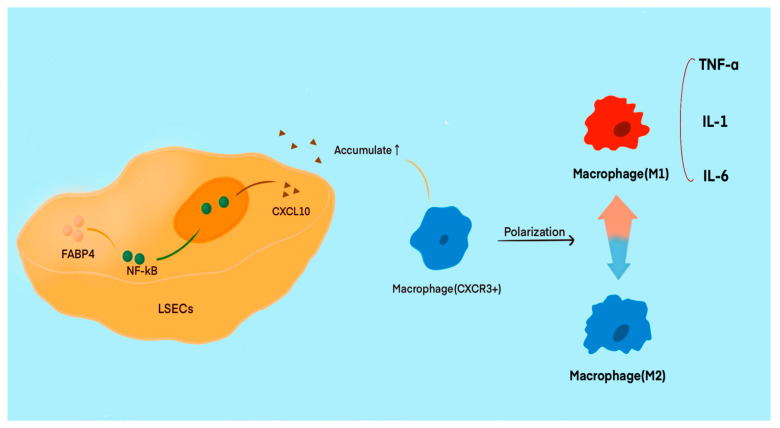
The increased expression of FABP4 in LSECs may represent a potential target for liver injury. Under certain conditions, the upregulated expression of FABP4 in LSECs activates the NF-κB/CXCL10 pathway, which leads to the recruitment of CXCR3+ macrophages and induces their polarization towards the M1 phenotype. This polarization leads to the release of a significant amount of proinflammatory mediators, such as TNF-α and IL-6, which continuously exacerbate the immune response. Conversely, the M2 phenotype of macrophages secretes anti-inflammatory factors, preventing excessive immune reactions.

Previous studies have indicated that LSECs play a crucial role in liver injury, especially those caused by chronic inflammation, such as non-alcoholic fatty liver disease and metabolic disorders. Immune-related factors are often the primary inducers of liver injury. FABP4, a member of the fatty acid-binding protein multigene family, significantly impacts the development of metabolic and inflammatory conditions in the body [104,105]. FABP4 plays a critical role in regulating inflammation and cell apoptosis during inflammatory responses. Recent studies have indicated an increased expression of FABP4 in LSECs, making it a potential target for liver injury. Under specific conditions, the upregulation of FABP4 in LSECs leads to its accumulation in the cytoplasm and can activate the NF-κB/CXCL10 pathway, thereby affecting immune regulation. FABP4 promotes the translocation of NF-κB/p65 from the cytoplasm to the nucleus, enhancing the transcription, expression, and secretion of CXCL10 [106]. The elevation of CXCL10 levels contributes to the recruitment of CXCR3+ macrophages and promotes their polarization towards the M1 phenotype (Figure 3). Macrophages, as crucial immune cells, play a significant role in various immune responses. Serving as key immunomodulators, macrophages exhibit different phenotypes involved in antigen presentation and other immune functions. The M1 phenotype produces proinflammatory mediators, such as TNF-α, IL-1, and IL-6, which act as important proinflammatory agents. Conversely, M2 phenotype macrophages secrete anti-inflammatory mediators, providing protection during inflammatory responses. These two phenotypes can switch in specific environments, triggering corresponding effects [107,108].

As an inflammatory state triggered by acute infection, LSECs may be involved in the immune response during sepsis. The roles of FABP4 and the NF-κB pathway in sepsis-related responses within LSECs require further investigation [109]. The persistent enhancement of the immune response may be a significant factor contributing to sepsis-associated multiple infections and intractable microcirculatory disturbances. Addressing the issue of immunosuppression could potentially offer a promising avenue for research aimed at improving survival rates in sepsis.

### 4.2. The Role of LSECs in Chronic Inflammation

Fatty liver disease is a common chronic non-alcoholic condition characterized by persistent inflammation, hepatocellular injury, and fibrosis. Alterations in the lobular structure of the liver can lead to hemodynamic disturbances. Consequently, even in the early stages of the disease, LSECs may suffer from ischemic injury and capillarization, resulting in the loss of their unique structure and function, despite the absence of significant inflammatory damage [110]. Animal studies have indicated that damaged and dysfunctional LSECs can release profibrotic molecules, such as TGF-β, forming a positive feedback loop during the fibrosis process. This leads to chronic inflammation, which drives the progression of fibrosis and ultimately results in portal hypertension [111]. As a specialized endothelial cell type, the damage to LSECs is critical for the progression of typical immune responses and the adverse effects resulting from functional impairment. This is important not only in acute inflammatory responses but also in chronic inflammation, where LSECs play a significant role.

### 4.3. Pathogenesis of Septic Coagulation Disease by LSECs

LSECs play a pivotal role in the development and progression of sepsis-associated coagulation disorders. Sepsis, a systemic inflammatory response syndrome triggered by infection, is often accompanied by coagulation system disorders, which may ultimately lead to disseminated intravascular coagulation (DIC) [112]. Under septic conditions, pathogenic microorganisms and their toxins (such as endotoxins) induce the release of inflammatory factors, resulting in damage and dysfunction of LSECs. Capillarization, cell swelling, and increased permeability occur in LSECs, leading to a decrease in normal anticoagulant function and further promoting coagulation reactions [113]. Furthermore, inflammatory cytokines such as TNF-α, IL-1β, and IL-6 stimulate LSECs to significantly upregulate the expression of tissue factor (TF). The binding of tissue factor to coagulation factor VII activates the coagulation cascade, resulting in the generation of large amounts of thrombin and fibrin, thereby accelerating thrombosis [114].

The anticoagulant function of LSECs is significantly inhibited during sepsis. Under normal conditions, LSECs exert their anticoagulant effect by expressing substances such as thrombomodulin (TM) [115], However, during sepsis, the expression of these anticoagulant factors decreases, impeding the activation of protein C and reducing the activity of antithrombin III, which further intensifies the coagulation process [116]. Simultaneously, the ability of LSECs to regulate fibrinolytic function is also affected. During the early inflammatory response, LSECs release a large amount of tissue plasminogen activator (tPA), promoting fibrinolysis [117], However, as inflammation progresses, the secretion of plasminogen activator inhibitor-1 (PAI-1) by LSECs increases significantly, inhibiting tPA activity and resulting in impaired fibrinolysis, persistent microthrombi formation, and accumulation [118]. Furthermore, LSECs play a bridging role between inflammation and coagulation responses. Inflammatory factors induce LSEC dysfunction and promote coagulation cascade reactions, while thrombin and fibrin generated during the coagulation process further activate LSECs to release proinflammatory factors. These pathological changes ultimately lead to organ microcirculatory dysfunction. In summary, LSEC dysfunction plays a central role in the pathogenesis of sepsis-related coagulation disorders. Inflammatory factors induce LSECs to upregulate tissue factor expression, initiating the extrinsic coagulation pathway while inhibiting anticoagulation and fibrinolysis functions, leading to thrombosis and triggering organ microcirculatory dysfunction. The interaction between inflammation and coagulation exacerbates the condition, which may ultimately develop into disseminated intravascular coagulation (DIC) and multiple organ failure.

## 5. Discussion

In the process of LPS clearance, there exists a synergistic effect between LSECs and KCs. These two types of cells collaborate through endocytosis and phagocytosis to eliminate invading endotoxins, thereby enhancing the efficiency of LPS clearance and reducing inflammatory responses. Enhancing the ability of LSECs to clear LPS can be considered a key strategy in regulating the progression and severity of sepsis. Conversely, dysfunction of LSECs may also be a critical factor in disease progression. Therefore, further investigation into the mechanisms of LSECs and their role in LPS clearance, as well as identification of the key steps and initiating factors in LPS clearance, can provide a scientific basis for reducing inflammatory responses during sepsis. A recent review on LPS clearance mentioned the dynamic balance between LPS clearance and LPS signaling [119]. Effective clearance of LPS in the body can alleviate the excessive secretion of inflammatory factors caused by subsequent LPS-related signaling pathways. However, excessive LPS signaling can lead to damaging effects and impact the function of clearance receptors. From this perspective, further research is still needed to elucidate the interaction between LPS clearance and signaling.

As a special type of endothelial cell, the immune function of LSECs is also of great exploration value. LSECs are involved in multiple links of the immune response. The relevant content is summarized in Table 1. LSECs recognize LPS through the expression of TLR4, activating both MYD88-dependent and -independent signaling pathways. This leads to the induction of molecular cascade reactions, such as NF-κB and MAPK, promoting the secretion of proinflammatory cytokines including TNF-α, IL-6, and IL-1β, and rapidly initiating immune defense responses. However, in sepsis, the excessive activation of TLR4 results in impaired LSEC function, increased endothelial barrier permeability, and exacerbated endotoxin circulation and multi-organ dysfunction. Thus, interventional measures targeting the TLR4 signaling pathway may emerge as novel strategies for the prevention and treatment of sepsis-associated liver injury. As atypical APCs, LSECs have garnered increasing attention for their immunomodulatory role in sepsis. During infection, LSECs upregulate the expression of MHC II, presenting antigens and activating T cells to regulate adaptive immune responses. Additionally, LSECs suppress T cell activation through the PD-L1 pathway, preventing excessive immune reactions and reducing early inflammatory damage in sepsis. However, persistent high expression of PD-L1 can lead to immunosuppression, increasing the risk of secondary infections. Targeting the PD-1/PD-L1 pathway has been shown to significantly improve survival rates in animal models of sepsis, suggesting its potential for clinical application in immunomodulation [120].

In addition, LSECs regulate the recruitment and activation of immune cells by secreting cytokines such as IL-6 and expressing adhesion molecules like ICAM-1 and VCAM-1 during inflammatory responses. IL-6, an important biomarker of sepsis, has a level closely correlated with the severity of the disease. LSECs promote the polarization of macrophages towards the M1 phenotype through chemokines like CXCL10, further exacerbating the inflammatory response. It is noteworthy that LSECs regulate the activation of TGF-β, promoting the differentiation of Tregs and maintaining immune homeostasis while suppressing excessive inflammatory responses [121]. However, with the progression of sepsis, the overactivation of Tregs may lead to immunosuppression, exacerbating secondary infections and organ damage in the later stages of sepsis. Therefore, balancing the LSEC–TGF-β–Treg axis is crucial for improving immune homeostasis in sepsis. FABP4 is an important potential target for regulating inflammation and apoptosis in various metabolic diseases and inflammatory processes. FABP4 affects immune processes through multiple pathways, including regulating cytokine secretion involved in proinflammatory responses when interacting with pathways such as NF-κB. Additionally, FABP4 participates in the development of organ dysfunction caused by inflammatory responses in sepsis. The consequences of proinflammatory responses include inflammatory mediator-induced endothelial cell injury, leading to irreversible organ dysfunction. The signaling pathways related to FABP4 may be key strategies to improve the pathological process of sepsis. Further research is needed to verify the role of LSECs and their immune function in this context.

The mechanism of action of LSECs in sepsis is complex and diverse, involving multiple aspects such as the TLR4 signaling pathway, PD-L1-mediated immune regulation, the secretion of cytokines like IL-6 and CXCL10, and the regulation of Tregs. Future research should focus on exploring targeted intervention strategies for key pathways including TLR4, PD-L1, and FABP4, aiming to protect the structural and functional integrity of LSECs and improve sepsis-related immune imbalance and organ dysfunction. A deeper understanding of the immune regulatory mechanism of LSECs in sepsis will provide a new theoretical basis and clinical direction for early intervention and precision treatment.

## Figures and Tables

**Table 1:** The immunomodulatory role of LSECs in the inflammatory response process.

Functional Module	Specific Role	Mechanisms and Involved Molecules	References
Antigen Presentation	- Cross-presentation of exogenous antigens to activate CD8+ T cells, clearing blood pathogens - Activation of NKT cells through lipid and glycolipid antigen presentation	- MHC-I cross-presentation of exogenous antigens (e.g., viral antigens) - CD1d presenting lipid/glycolipid antigens to NKT cells - Involvement of pattern recognition receptors (PRRs), scavenger receptors (SRs), and mannose receptors	[50,51,52,53,54,55,56]
Spatial Polarization	- Regulates spatial distribution of immune cells to confine pathogens - Establishes immune spatial polarization in liver sinusoids	- MYD88-dependent signaling pathway - Chemokine formation and extracellular matrix remodeling - Coordination of immune cell localization to restrict bacterial activity	[57,58,59,60]
Cell Recruitment	- Promotes recruitment and accumulation of leukocytes and lymphocytes in the liver during inflammation - Enhances immune cell interaction and inflammatory response	- Differential expression of adhesion molecules (ICAM-1 and VCAM-1) - Chemokines such as CXCL16 and CXCL10 for CD8+ T cell recruitment - Interaction with NK cells and modulation of cytokine production	[61,62,63,64,65,66]
Cytokine Expression	- Regulates production of pro-inflammatory cytokines (e.g., IL-6, TNF-α) - Influences the immune response and sepsis progression	- Activation by LPS via TLR4 signaling - Expression of CD54 and CD106 - Involvement of TGF-β, IL-12, and IL-18, modulating NK cell activity and immune response	[68,69,70,71,72]
PD-L1 Pathway	- Modulates immune suppression and influences sepsis progression - Inhibits lymphocyte apoptosis and reverses monocyte dysfunction	- PD-1/PD-L1 pathway mediates immune suppression - Upregulation of PD-L1 during sepsis - Interaction with PD-1+ Kupffer cells (KCs) influencing liver injury	[73,74,75,76,77,78,79,80]
Regulatory T Cell Differentiation	- Promotes generation and function of regulatory T cells (Tregs) - Maintains immune tolerance and prevents autoimmune liver damage	- GARP-mediated activation of latent TGF-β - TGF-β-induced Smad phosphorylation and nuclear translocation - Regulation of Treg differentiation and inhibitory cytokine production (IL-10 and TGF-β)	[81,82,83,84,85,86,87,88]
TLR4 Expression	- Regulates LPS response and pro-inflammatory cytokine production - Enhances local immunity and inflammatory response	- TLR4/MYD88-dependent pathway activating NF-kB and MAPK signaling - TRIF pathway promoting selectin, ICAM-1, and VCAM-1 expression - Modulation of LSEC permeability and structural function	[89,90,91,92,93,94,95,96,97,98,99,100]
FABP4 Influence on Metabolism and Inflammation	- Regulates metabolism and inflammatory responses - Influences macrophage polarization (M1/M2)	- Upregulation of FABP4 activates NF-κB/CXCL10 pathway - Recruitment of CXCR3+ macrophages and polarization to M1 phenotype - Interaction with pro-inflammatory mediators (TNF-α, IL-1, and IL-6)	[101,102,103,104,105,106]

## Data Availability

No new data were created or analyzed in this study.

## Abbreviations

The following abbreviations are used in this manuscript:

LSECs	Liver sinusoidal endothelial cells
APCs	Antigen-presenting cells
KCs	Kupffer cells
LPS	Lipopolysaccharide
SR	Scavenger receptor
MR	Mannose receptor
SIC	Small immune complexes
HDL	High-density lipoprotein
PD-L1	Programmed death ligand-1
MHC II	Major histocompatibility complex class II
PRRs	Pattern recognition receptors
MYD88	Myeloid differentiation primary response 88
ICAM-1	Intercellular adhesion molecule 1
VCAM-1	Vascular cell adhesion protein 1
CXCR16	CXC chemokine ligand 16
CXCR6	CXC chemokine receptor 6
CXCL10	CXC chemokine ligand 10
TNF-α	Tumor necrosis factor-α
IL	Interleukin
TLR4	Toll-like receptors 4
TGF-β1	Transforming growth factor-beta 1
NK	Nature killer
CLP	Cecal ligation and puncture
GARP	Glycoprotein A Repetitions Predominant
Tregs	Regulatory T cells
FABP4	Fatty acid-binding protein 4
DIC	Disseminated intravascular coagulation
TF	Tissue factor
TM	Thrombomodulin
tPA	Tissue plasminogen activator
PAI-1	Plasminogen activator inhibitor-1
